# Editorial: Exploring wood structure and tree-ring dynamics in ecological research

**DOI:** 10.3389/fpls.2025.1736889

**Published:** 2025-11-27

**Authors:** Alma Piermattei, Ignacio García-González, Fabio Gennaretti, Marta Górska, Luis Silva, Alan Crivellaro

**Affiliations:** 1 Department of Agricultural, Forest and Food Sciences, Università degli Studi di Torino, Grugliasco, Italy; 2 Forest Biometrics Laboratory, Faculty of Forestry, “Stefan cel Mare” University of Suceava, Suceava, Romania; 3 Ignacio García-González, Institute of Agrarian Biodiversity and Rural Development (IBADER) - Department of Botany, Universidade de Santiago de Compostela, Lugo, Spain; 4 Department of Agricultural, Food and Environmental Sciences, Università Politecnica delle Marche, Ancona, Italy; 5 Faculty of Forestry and Wood Technology, Poznań University of Life Sciences, Poznań, Poland; 6 Department of Biology, Universidade dos Açores, Ponta Delgada, Portugal

**Keywords:** quantitative wood anatomy (QWA), tree-ring dynamics, dendrochronology, ecological resilience, climate reconstruction

Wood structure and tree-ring patterns are among the most powerful natural archives for reconstructing past climates and understanding ecological processes (Büntgen et al., 2020). From cell dimensions to intra-annual growth anomalies, these microscopic details offer insights into how trees respond to shifting environmental conditions (Bräuning et al., 2016). At a time when global change is intensifying, such records are crucial for predicting plant resilience, informing ecosystem and landscape conservation efforts, and supporting sustainable resource management. The aim of the Research Topic “Exploring Wood Structure and Tree-Ring Dynamics in Ecological Research” was to bring together studies that integrate advances in wood anatomy and tree-ring dynamics to deepen our understanding of ecological processes and plant responses under environmental change. The eight contributions assembled here collectively showcase both the ecological breadth and the methodological depth of contemporary wood anatomical research (**Figure 1**). The contributions span a wide geographic range, from the Mediterranean and subtropical Asia to North America and the northern treeline, and also advance methodological tools that enhance quantitative wood anatomical analyses. Together, they highlight both the ecological scope and the technical depth of contemporary research on wood anatomy.

**Figure 1:**
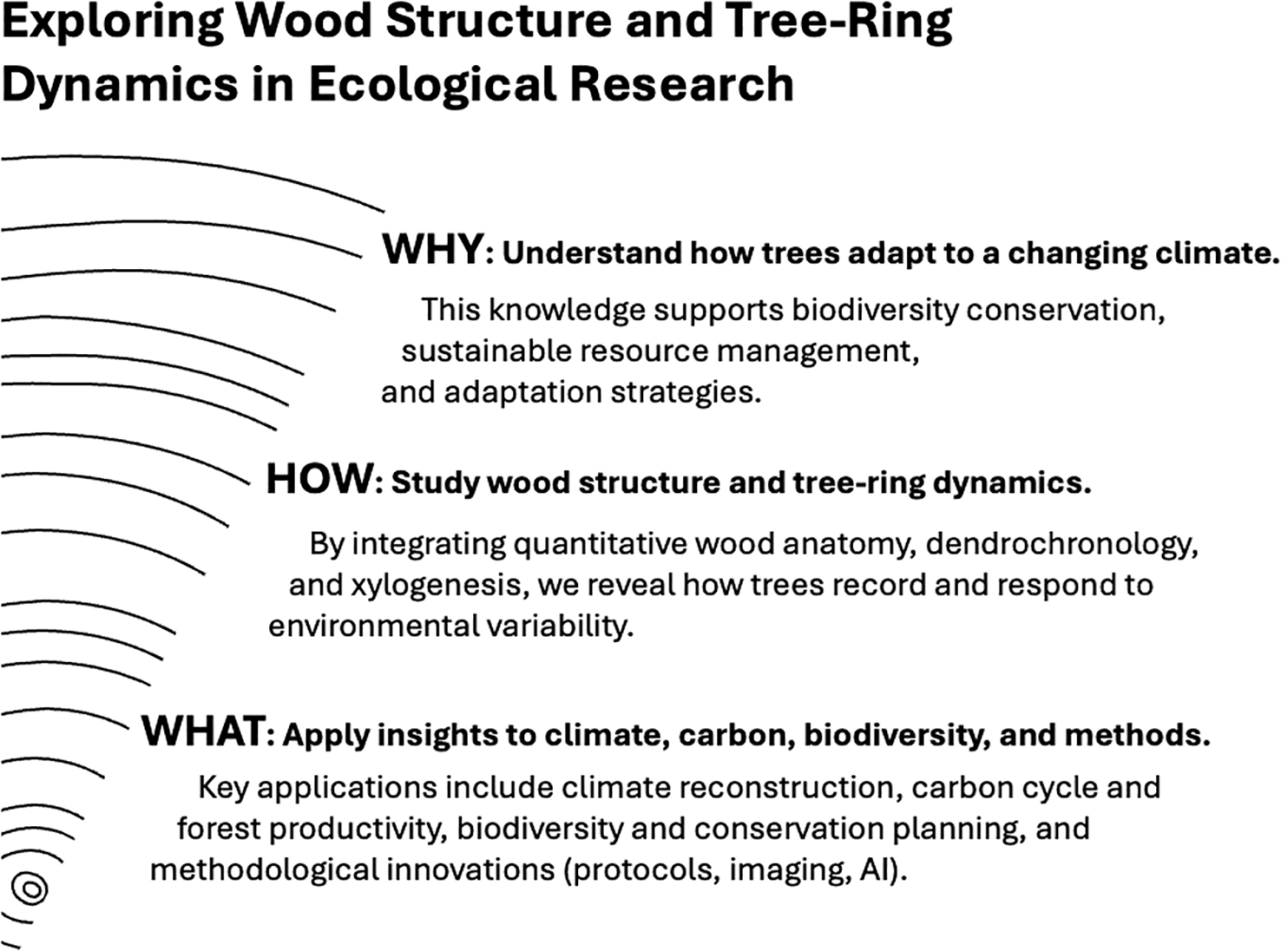
Conceptual framework of the Research Topic “Exploring Wood Structure and Tree-Ring Dynamics in Ecological Research”. The diagram illustrates the progression from WHAT (applications and insights: climate reconstruction, carbon cycle and productivity, biodiversity and conservation), through HOW (wood structure and tree-ring dynamics via quantitative wood anatomy, dendrochronology, and xylogenesis, including methodological innovations), to WHY (understanding ecological processes and plant resilience under global change).

## Climate and growth responses across ecosystems

Some studies in this Research Topic illustrate how wood anatomical traits capture fine-scale climatic variability across diverse environments.

In the eastern Mediterranean, Mdawar et al. provide the first quantitative wood anatomical study of *Juniperus excelsa* in Lebanon, showing that lumen diameter is tightly linked to May precipitation and temperature. Their results suggest that declining anatomical traits since the 1990s reflect intensifying drought stress, pointing to the potential of this species as a hydroclimate archive in a water-scarce region. At the opposite latitudinal extreme, Buchwal et al. examine “blue rings” in trees and shrubs from the northern treeline of Fennoscandia. These anatomical anomalies, resulting from incomplete lignification under cold conditions, provide intra-annual markers of early- and late-summer cooling events, refining temperature reconstructions in a region central to climate studies.

Between these climatic poles, two further contributions highlight how growth dynamics respond to seasonal water availability. In humid subtropical China, Wang et al. demonstrate that late-summer rainfall strongly influences intra-annual density fluctuations (IADFs), with drought years producing rare unimodal growth patterns and wetter summers enhancing latewood formation. In boreal Canada, He et al. demonstrate how warming temperatures accelerate the onset of xylogenesis and increase cell production in balsam fir, projecting a potential 85% increase in xylem formation by 2080, with profound implications for carbon sequestration in northern forests.

Finally, Andrés-Hernández and Rodríguez-Ramírez shift the focus to tropical montane cloud forests of Mexico, ecosystems characterised by mild temperatures, persistent humidity, and frequent fog immersion, in contrast to the limiting environments that dominate most wood anatomical studies. They analyse a set of broadleaf diffuse-porous tree species, a group still underrepresented in quantitative wood anatomical research, integrating both wood and leaf traits to explore functional adjustments to climatic variation. Their results reveal significant interspecific divergence in vessel architecture and leaf venation patterns, highlighting how hydraulic efficiency and safety trade-offs underpin adaptive strategies to microclimatic stress. This work expands the scope of wood anatomical ecology to include humid, non-limiting environments and underscores the functional diversity of species in ecosystems highly vulnerable to climate change.

Together, these five studies illustrate the global relevance of wood structure as a climate-sensitive archive, capturing signals of water stress, temperature extremes, and growth resilience across diverse environments, including Lebanese juniper stands, Chinese conifer plantations, boreal fir forests, broadleaf tropical cloud forests, and the Arctic treeline. These signals would otherwise be difficult to detect using classical dendrochronological approaches.

## Methodological and technological advances

Complementing these ecological insights are three articles that advance the methodological foundations of quantitative wood anatomy.

A practical bottleneck in the field is the preparation of consistent, high-quality anatomical sections. Fonti et al. address this by providing a detailed protocol for sectioning wood samples, ensuring reproducibility across laboratories and enabling robust long-term anatomical chronologies.

Peters et al. tackle another critical step in quantitative wood anatomy, image acquisition, by systematically comparing surface imaging, microsections, and X-ray computed tomography. Their results show broad comparability across methods for hydraulic parameters, validating more accessible approaches while also clarifying their limitations, particularly for density estimates.

Looking ahead, Katzenmaier et al. present ROXAS AI, a machine learning framework tested on challenging shrub cross-sections. The approach substantially improves detection accuracy and provides uncertainty estimates, paving the way for automated, scalable analysis across multiple species.

These advances, from protocols and imaging to artificial intelligence, are critical steps in reducing the time and expertise required for wood anatomical studies, thus enabling broader adoption and larger-scale syntheses.

## Outlook

Looking ahead, three directions appear especially promising. First, integrating quantitative anatomy with other proxies, such as isotopes, remote sensing, xylogenesis, and phenology, will create more comprehensive ecological reconstructions. Second, expanding research into underrepresented ecosystems and taxa will capture the full spectrum of plant strategies and vulnerabilities. Third, collaborative development of open datasets and analytical tools will accelerate and standardise comparative studies and meta-analyses, strengthening the role of wood anatomy in global change research.

The eight contributions to Exploring Wood Structure and Tree-Ring Dynamics in Ecological Research together demonstrate both the enduring relevance and the expanding horizons of wood anatomical studies. From practical protocols to AI-assisted tools, and from drought-stressed junipers in Lebanon or multiple angiosperms from humid tropical Mexico, to temperature-sensitive blue rings at the Arctic treeline, this Research Topic underscores the versatility of wood structure as a lens onto ecological and climatic processes.

As Topic Editors, we hope this Research Topic not only advances ongoing debates but also inspires future collaborations across disciplines, methodologies, and regions. Wood, in its intricate structure, continues to tell the story of trees and ecosystems; our task as scientists is to refine the ways we read it.
